# Congenital Anomalous Origin of Coronary Artery Disease in Children With Syncope: A Case Series

**DOI:** 10.3389/fped.2022.879753

**Published:** 2022-07-05

**Authors:** Yumeng Gao, Qingyou Zhang, Yan Sun, Junbao Du

**Affiliations:** Department of Pediatrics, Peking University First Hospital, Beijing, China

**Keywords:** syncope, pediatric, coronary artery disease, anomalous aortic origin of a coronary artery, bezold-jarisch reflex

## Abstract

**Objective:**

This study is aimed to analyze the characteristics of congenital anomalous origin of coronary artery in pediatric patients with syncope.

**Methods:**

A total of eight patients were retrospectively analyzed from August 2018 to August 2020 who were admitted to the Peking University First Hospital with the complaint of syncope and were diagnosed with congenital coronary artery disease.

**Results:**

In total, eight patients were included in the study with a median age of 12.5 ± 2.7 (8–16) years. In total, four of them were males, and four were females. Six of the eight patients were diagnosed with right anomalous coronary artery from the opposite sinus (R-ACAOS), while two patients were diagnosed with left anomalous coronary artery from the opposite sinus (L-ACAOS). The most frequent inducement was exercise, and the commonest prodromes were dizziness and blurred vision. Serum cardiac markers and exercise electrocardiography test (EET) were normal in seven of the patients. The majority of cases had abnormal electrocardiograms (ECGs), but only two of them manifested elevated/depressed ST-T segments. In total, seven patients had positive head-up tilt test (HUT). Echocardiography and coronary artery computed tomography angiography (CTA) were performed to aid the diagnosis. Coronary unroofing procedures were conducted in four patients, and none of them reported syncope after the surgery. The other four patients received routine medical treatment for vasovagal-like syncope. In total, two patients out of them became asymptomatic, and in the other two patients, episodes of syncope were reduced, but they still required medical treatment.

**Conclusion:**

Congenital coronary artery anomalies in children with syncope need prompt attention. Though ECG and echocardiography are the common methods for investigating cardiac syncope, they have limited ability to find coronary artery anomalies. When coronary artery anomalies are suspected, coronary CTA should be considered.

## Introduction

Syncope is defined as a sudden loss of consciousness caused by global hypoperfusion of the brain (1, 2). Its incidence is 15–25% in children, predominantly occurring in female child (3). The relapse rate of syncope is as high as 33–51% within 5 years (3). Etiologies of transient loss of consciousness in the young include vasovagal syncope (70–80%), psychogenic and unexplained diseases (20%), and cardiovascular diseases (2–3%) (4). Though cardiac syncope comprises a small proportion of the pediatric syncope, it is often associated with high mortality due to sudden cardiac death (SCD), and hence requires urgent assessment and treatment (5). Cardiac diseases leading to syncope include arrhythmias, cardiac tumors, and structural heart diseases. Structural heart disease is classified into coronary artery anomalies, valvular heart disease, hypertrophic obstructive cardiomyopathy, idiopathic pulmonary hypertension, etc (6, 7). After combining thorough history, physical examination, and ECGs, nearly 50% of the cases can be diagnosed (5). Moreover, echocardiography and Holter-monitoring can contribute to confirming the etiological diagnosis of cardiac syncope. However, some coronary artery anomalies with no special medical history or abnormal imaging results may be difficult to diagnose through a routine approach. To improve the evaluation and management of the disease, we present the case series of eight children suffering from congenital coronary artery disease with syncope.

## Materials and Methods

We retrospectively reviewed all the 371 pediatric patients hospitalized with the chief complaint of syncope from August 2018 to August 2020 and found that eight (2.2%) patients were diagnosed with congenital coronary artery disease through coronary CTA or coronary angiography. Criteria used for coronary CTA were (1) high probability of cardiogenic syncopes, such as exercise-related syncope and syncope with a previous history of Kawasaki disease; (2) children with symptoms suggestive of coronary artery disease, such as syncope with chest pain. Personal details and clinical history of the patients were recorded, including age, gender, clinical manifestations, laboratory examinations (e.g., serum CK–MB, cTnI, and BNP), ECG, echocardiography, coronary CTA, etc. The follow-up period was 12–34 months (Table 2). Descriptive analysis was used to analyze the data.

## Results

### Clinical Characteristics

In total, eight patients were included in the study with a median age of 12.5 ± 2.7 (8–16) years. In total, four of them were men, and four were women. Six of them were diagnosed with the right anomalous coronary artery from the opposite sinus (R-ACAOS), while two were diagnosed with the left anomalous coronary artery from the opposite sinus (L-ACAOS). The clinical history and manifestations are shown in Table 1. The triggers of syncope were exercise (three cases) and postural change (three cases). However, no obvious trigger was found in four patients. Six children complained of prodromes before syncope, predominantly dizziness and blurred vision. Concomitant symptoms such as incontinence and limb twitching were not reported. The duration of syncope was usually short, lasting from a few seconds to 3 min. A family history for syncope was present in one patient.

**TABLE 1:** Clinical history and manifestations.

Patient number	Gender	Age (year)	Course of illness (month)	Number of episode	Syncope during exercise	Syncope after postural change	Precursor	Duration of syncope (min)	Family history
1	F	10	24	1	-	+	+	1	-
2	M	14	4	5	-	-	+	<1	-
3	F	15	12	2	+	+	+	2	-
4	M	11	84	20	-	-	+	<1	+
5	M	14	6	3	-	-	-	<1	-
6	F	16	72	2	+	+	+	<1	-
7	M	8	2	1	-	-	-	<1	-
8	F	10	12	2	+	-	+	3	-

*Gender: F, female, M, male.*

**TABLE 2:** Laboratory and imaging findings, treatment, and prognosis.

Patient number	Myocardial necrosis biomarkers	ECG/Holter ECG	HUT	Echo	Coronary artery CTA/Angiography	Treatment	Follow-up period (month)	Prognosis
1	-	Single premature ventricular contraction	POTS	Tiny branch of coronary artery	Interarterial R-ACAOS with no obvious stenosis	Beta-blocker	21	Less syncope
2	-	ST-T elevated	POTS	Tricuspid regurgitation	Interarterial R-ACAOS with proximal RCA stenosis	Coronary unroofing surgery	34	No symptom
3	-	Single premature ventricular contraction	POTS	L-ACAOS	Interarterial L-ACAOS with proximal LCA stenosis	Coronary unroofing surgery	27	No symptom
4	-	Normal	POTS	Left superior vena cava	Interarterial R-ACAOS with no obvious stenosis	Functional exercise, ORS	24	No symptom
5	-	Sinus arrest	OHT	Left atrium and ventricle enlargement	Interarterial R-ACAOS with proximal RCA stenosis	Functional exercise, ORS	23	No symptom
6	-	Left axis deviation	VVS	Tricuspid regurgitation	Interarterial R-ACAOS with proximal RCA stenosis	Alpha-1 adrenergic agonist	26	Less syncope
7	-	QTc 0.50 s	POTS	Normal	Interarterial R-ACAOS with proximal RCA stenosis	Coronary unroofing surgery	22	No symptom
8	+	ST-T depressed	/	Left atrium enlargement	Interarterial L-ACAOS with proximal LCA stenosis	Coronary unroofing surgery	12	No symptom

*ECGs, electrocardiograms; HUT, head-up tilt test; CTA, computed tomography angiography; POTS, postural tachycardia syndrome; OHT, orthostatic hypertension; VVS, vasovagal-like syncope; R-ACAOS, right anomalous coronary artery from the opposite sinus; L-ACAOS, left anomalous coronary artery from the opposite sinus; Echo, echocardiography; RCA, right coronary artery; LCA, left coronary artery; ORS, oral rehydration salt.*

### Serum Cardiac Markers

Myocardial necrosis biomarkers were measured routinely in all the 371 patients admitted to our hospital. The serum measurements were done 0–2 days after the syncope in all the eight patients. Serum cardiac markers (CK, CK–MB, and cTnI) were normal in seven patients. Myocardial necrosis biomarkers were significantly elevated in one patient (CK 1789IU/l, CK-MB 140IU/l, and cTnI 9.92 ng/ml).

### Electrocardiograms

**FIGURE 1:**
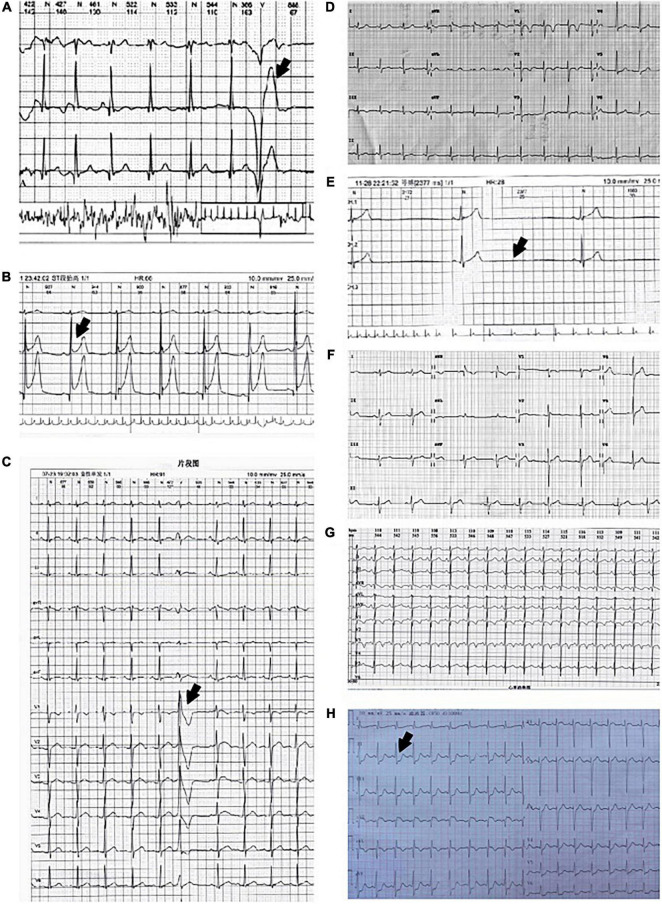
Electrocardiogram (ECG)/Holter ECG of eight patients. **(A)** Single premature ventricular contraction in Patient 1. **(B)** ST-T elevated in patient 2. **(C)** Single premature ventricular contraction in patient 3. **(D)** Normal ECG of patient 4. **(E)** Sinus arrest in patient 5. **(F)** Left axis deviation in patient 6. **(G)** QTc 0.50 s in patient 7. **(H)** ST-T depressed in patient 8.

All the patients underwent routine 12-lead ECG and 24-h ambulatory electrocardiogram (Holter), and abnormal ECGs were recorded in most patients (Figure 1 and Table 2). The abnormalities included single premature ventricular contraction in two cases, ST-T changes in two cases, sinus arrest in one case, prolonged QT interval in one case, and axis deviation in 1 case. EET was conducted in six cases revealed no other abnormal results except abnormal ECG at rest.

### Head-Up Tilt Test

Head-up tilt test was conducted on seven patients. They were placed in the upright position, tilted upward at an angle of 60° and supine position, with simultaneous monitoring of heart rate, blood pressure, and ECG (8). All of the seven cases had positive findings. In total, five cases were found to have postural tachycardia syndrome (POTS), one case had orthostatic hypertension, and one case had vasovagal-like syncope (vaso-inhibitory type) (Table 2).

### Echocardiography

Echocardiography was done in all the cases, two of them were found to have coronary artery anomalies, and two had left atrium and/or ventricle enlargement.

### Coronary Computed Tomography Angiography

**FIGURE 2:**
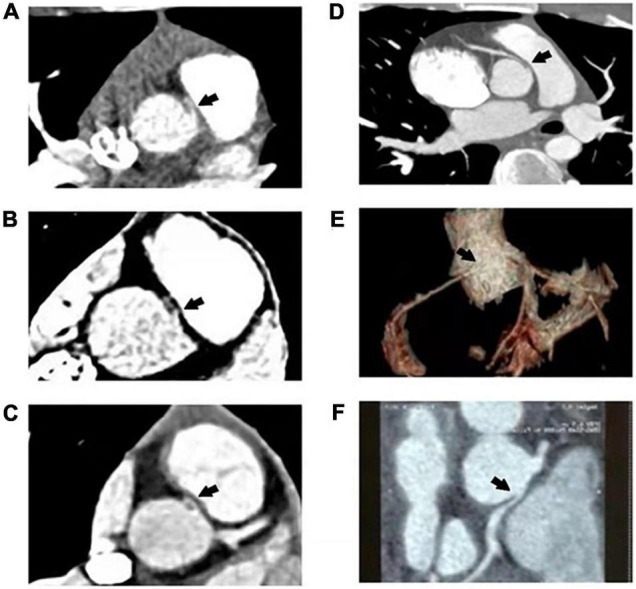
Computed tomography angiography (CTA) of six patients. Black arrows indicate the interarterial course between the aorta and pulmonary artery. **(A)** Interarterial R-ACAOS with no obvious stenosis (Patient 1). **(B)** Interarterial R-ACAOS with proximal RCA stenosis (Patient 2). **(C)** Interarterial R-ACAOS with no obvious stenosis (Patient 4). **(D)** Interarterial R-ACAOS with proximal RCA stenosis (Patient 6). **(E)** Interarterial R-ACAOS with proximal RCA stenosis (Patient 7). **(F)** Interarterial L-ACAOS with proximal LCA stenosis (Patient 8).

Coronary CTA was conducted to characterize coronary artery disease and detect coronary artery stenosis. From 1 August 2018 to 1 August 2020, coronary CTAs were performed on 74 patients in our hospital, and eight of them were diagnosed with AAOCA. We found that all the eight patients had intramural segments and different degrees of coronary artery narrowing (Figure 2 and Table 3). The ostial type was a separate ostium, and the take-off angle was less than 45° in all the cases (Table 3).

**TABLE 3:** Coronary artery computed tomography angiography (CTA).

Patient number	Coronary artery CTA	Intramural segment length (mm)	Proximal vessel morphology	Take off angle	Ostial type
1	Interarterial R-ACAOS with no obvious stenosis	2	Normal	<45°	Separate ostium
2	Interarterial R-ACAOS with proximal RCA stenosis	5	Oval	<45°	Separate ostium
3	Interarterial L-ACAOS with proximal LCA stenosis	3	Oval	<45°	Separate ostium
4	Interarterial R-ACAOS with no obvious stenosis	2	Normal	<45°	Separate ostium
5	Interarterial R-ACAOS with proximal RCA stenosis	5	Oval	<45°	Separate ostium
6	Interarterial R-ACAOS with proximal RCA stenosis	8	Oval	<45°	Separate ostium
7	Interarterial R-ACAOS with proximal RCA stenosis	3	Slit-like	<45°	Separate ostium
8	Interarterial L-ACAOS with proximal LCA stenosis	2	Oval	<45°	Separate ostium

*CTA, computed tomography angiography; R-ACAOS, right anomalous coronary artery from the opposite sinus; L-ACAOS, left anomalous coronary artery from the opposite sinus; RCA, right coronary artery; LCA, left coronary artery.*

### Treatment and Prognosis

Coronary unroofing procedures were performed on four patients (patient number 2, 3, 7, and 8), and none of the patients complained of syncope after the surgery. The rest of the four patients received treatment, such as functional exercise, oral rehydration salts (ORSs), beta-blocker, and alpha-1 adrenergic agonists. All the symptoms resolved in two patients (patient number 4 and 5), and they stopped treatment. The other two patients (patient number 1 and 6) suffered from fewer syncopal episodes than before and still needed medication (Table 2).

## Discussion

Though syncope caused by coronary artery disease among children has a low incidence, it poses a significant risk to their health. Anomalous aortic origin of a coronary artery (AAOCA) is one of the most common congenital coronary artery anomalies and can manifest as syncope in children. AAOCA is the second most common cause of sudden cardiac death (SCD) in young athletes (9). From 1 August 2018 to 1 August 2020, there were 371 children admitted to our hospital with syncope as the chief complaint, and 8 (2.2%) were diagnosed with congenital coronary artery disease. All of them were found to have AAOCA, and the incidence is higher than in the newborns (0.64%) (10) or in the asymptomatic children (0.17%) (11). Thus, syncope is an important clinical symptom of congenital coronary disease in children.

Anomalous aortic origin of a coronary artery can further be classified into five subtypes: interarterial, subpulmonic (intraconal or intraseptal), pre-pulmonic, retroaortic, and retrocardiac (12). L-ACAOS is generally associated with a high risk of SCD, and the benefits of revascularization in L-ACAOS patients likely outweigh the risks (12, 13). The study by Cheezum reported that R-ACAOS patients with interarterial compression suffered from more frequent syncopal episodes and chest pain than AAOCA without interarterial compression (3, 12). In another study by Kaushal (13), symptomatic patients with AAOCA had a longer intramural course than asymptomatic patients with AAOCA. These findings suggest that the appearance of symptoms is affected by the degree to which the ectopic artery is compressed. In total, eight patients in our study were diagnosed with AAOCA (interarterial type) with different levels of interarterial compression. Moreover, all of them were symptomatic. Studies have revealed that clinical manifestations of AAOCA vary from asymptomatic, palpitation, chest discomfort, cardiac syncope, and acute myocardial infarction to sudden death (12, 14). Whether there is a positive correlation between the severity of symptoms and the degree of coronary compression has not been elucidated.

Exercise stimulates myocardial contraction, thus, compressing the intramural vessels. Compressed coronary artery leads to ischemia of the heart conduction system and cardiomyocytes, resulting in arrhythmia and decreased myocardial contractility, respectively. Consequently, cardiac ejection fraction decreases, and hence, blood flow to the brain is diminished. This results in a syncopal attack. This pathogenesis may be accountable for the syncope during the exercise in three of the patients in our study. Syncope during exercise is an indicator of coronary disease.

However, three patients suffered from syncope after a sudden postural change, and seven patients had positive HUT findings, suggestive of vasovagal-like syncope. Although clinical features suggest vasovagal-like syncope, it is essential to rule out cardiac syncope before the diagnosis is confirmed. Further investigation and follow-up are essential in such cases (15–17).

Electrocardiogram changes in AAOCA are also significant in diagnosing interarterial compression. There may be no typical characteristics at rest, while short-lived myocardial ischemia caused by compressed coronary artery after exercise may appear as the abnormal QRS wave and the change of ST-T in ECG. Severe or continued compression also results in the elevation of myocardial necrosis biomarkers. So, when increased myocardial necrosis biomarkers or depressed/elevated ST-T in ECG are observed, especially after exercise in a child with syncope and AAOCA, it is strongly suggestive of syncope due to AAOCA. But even in the absence of the elevated myocardial necrosis biomarkers or changed ST-T in ECGs, the possibility of cardiac syncope cannot be excluded. The normal levels of myocardial necrosis biomarkers and the normal EET findings in our study may be due to very mild coronary artery compression, transient coronary stenosis, and the fact that these tests were not conducted during the ischemia. Two patients (the third and eighth) suffered from syncope during exercise, and we found that both of them suffered from interarterial L-ACAOS with proximal LCA stenosis. Considering the potential risks associated with EET, we did not recommend EET in these two cases. Some studies demonstrate that a normal EET result can not preclude SCDs (18). For those cases with strong evidence of cardiac ischemia, more sensitive and specific methods such as stress echocardiography or nuclear myocardial perfusion imaging may be necessary. Echocardiography is a common modality to evaluate suspected or confirmed cardiac disease as an inexpensive, efficient, non-invasive, and widely available modality. However, echocardiography plays a limited role in evaluating AAOCA due to its minimal ability to visualize surrounding structures, limited dynamic imaging, low-spatial resolution, dependence on body habitus, and operator competence (12). CTA, which is the Class I-indicated test used to image AAOCA, is still a preferred modality (18). Coronary CTAs were performed on 74 patients in our hospital, and eight (10.8%) of them were diagnosed with AAOCA. It signifies that coronary CTA plays a vital role in diagnosing syncope caused by coronary disease.

The management of AAOCA depends on the symptoms or diagnostic evidence of coronary ischemia due to the anomalous coronary artery. Surgery is the Class I-indicated treatment in such cases in R-ACAOS or L-ACAOS (18–20). In L-ACAOS, even without ischemic symptoms or diagnostic evidence, surgery is recommended (Class IIa) because it is associated with a higher risk of SCD (18–20). However, in patients with R-ACAOS without ischemic symptoms or diagnostic evidence, surgical intervention is not proven to alter the risk of SCD. Thus, the management option for these patients is still controversial and watchful waiting, and also surgery is Class IIb-indicated (18–20). In our study, two patients with L-ACAOS and two patients with symptomatic R-ACAOS underwent coronary unroofing surgery and were relieved of syncope after the surgery. The other four patients with symptomatic R-ACAOS also had the indication for surgical treatment, but they denied surgery. These patients were observed on follow-up visits and received conservative treatment, though the possibility of opting for surgery in the future was explained to them. We observed that BJR activation, one of the main mechanisms for vasovagal syncope, could also exist in AAOCA. Thus, the syncope attributable to AAOCA might be associated not only with decreased coronary flow but also with vasovagal-like syncope. In our study, two patients of AAOCA with proximal coronary stenosis receiving treatment for vasovagal-like syncope reported reduced or no syncopal episodes during the follow-up period. This suggests that vasovagal response may also be an etiology for syncope.

There are also some limitations in our study. AAOCA may be underestimated in our study due to a limited number of patients undergoing echocardiography and CT scan. We propose that the reason for the low-diagnosis rate of echocardiography is that the routine echocardiography does not include coronary examination in some hospitals of China. One of the primary significances of this study is to emphasize that in addition to routine echocardiography, further attention should be paid to the detection of coronary malformations in children with syncope, and pediatricians and sonographers should be vigilant about it. In addition, another limitation is that we could not provide the cross-sectional imaging of all the patients with relative 3D reconstruction. Moreover, the follow-up period in our study was not long enough for a valid long-term assessment. The increased frequency of the presence of AAOCA is very low. Therefore the sample size of the cases in the study group should be much larger to obtain statistically significant differences in comparison with the historical group of other authors. Furthermore, it should be stressed that the patients with the greatest risk of death with the left main artery coming from the pulmonary trunk were not observed in the study likely due to the small sample size.

Therefore, we conclude that more attention should be paid to congenital coronary disease in children with syncope. ECG and echocardiography, as the common methods for investigating cardiac syncope, may have limited ability to find coronary artery anomalies. Patients with positive HUT results should not be simply diagnosed with vasovagal syncope. In the cases of suspected cardiac syncope, ruling out coronary artery anomalies through coronary CTA is imperative. In AAOCA, L-ACAOS can be symptomatic and need close monitoring and even surgery, but in R-ACAOS, the etiology of syncope needs to be thoroughly evaluated.

## Data Availability Statement

The original contributions presented in this study are included in the article/supplementary material, further inquiries can be directed to the corresponding author.

## Ethics Statement

The studies involving human participants were reviewed and approved by the Peking University First Hospital Ethics Committee. Written informed consent to participate in this study was provided by the participants’ legal guardian/next of kin. Written informed consent was obtained from the legal guardian/next of kin for the publication of any potentially identifiable images or data included in this article.

## Author Contributions

YG: design and implementation of experiments, data collection and analysis, statistical analysis, and drafting of the article. QZ: design of experiments, data interpretation and analysis, statistical analysis, guidance of the article drafting, and critical review. YS: implementation of experiments, data collection and analysis, statistical analysis, drafting of the article, and supporting contribution. JD: design of experiments, data interpretation and analysis, statistical analysis, guidance of the article drafting, critical review, and supporting contribution. All authors contributed to the article and approved the submitted version.

## Conflict of Interest

The authors declare that the research was conducted in the absence of any commercial or financial relationships that could be construed as a potential conflict of interest.

## Publisher’s Note

All claims expressed in this article are solely those of the authors and do not necessarily represent those of their affiliated organizations, or those of the publisher, the editors and the reviewers. Any product that may be evaluated in this article, or claim that may be made by its manufacturer, is not guaranteed or endorsed by the publisher.
